# Commentary on: Global Expert Opinion on Cryolipolysis Treatment Recommendations and Considerations: A Modified Delphi Study

**DOI:** 10.1093/asjof/ojac016

**Published:** 2022-03-10

**Authors:** David P Rapaport

**Affiliations:** Department of Plastic Surgery, Tel Aviv Sourasky Medical Center, Tel Aviv, Israel

See the Original Article here.

“A consensus means that everyone agrees to say collectively what no one believes individually”—Abba Eban

In this level 5 study,^1^ a panel of 11 global experts in cryolipolysis were assembled by Allergan (an Abbvie Company, Irvine, CA), the study sponsor and owner of CoolSculpting, with the goal of reaching consensus on several aspects of CoolSculpting treatment, including defining outcomes, patient evaluation and selection, treatment protocols by body region, management of patient satisfaction, and management strategies for adverse events (AEs). Five of the panelists were plastic surgeons, 4 were dermatologists, and the other 2 were referred to as experts in cosmetology and aesthetics. The study was constructed as a modified Delphi study aimed at achieving consensus by first exploring the panel’s opinions on a 39-question survey. As opposed to a traditional Delphi study^2^ in which all panelists remain anonymous to one another and sessions do not have a predetermined duration or quantity, this study had only 2 surveys, with loss of anonymity occurring at a single virtual meeting of the panelists midway in the study. The second survey consisted of 61 statements designed to drive consensus wherein panelists offered their responses on a 5-point scale (strongly disagree, disagree, neither agree nor disagree, agree, and strongly agree). Consensus was defined as complete when all 11 panelists agreed, strong when at least 9 were in agreement, and moderate when 7 of 11 panelists (63.6%) were in agreement (Video).

Although cryolipolysis is the leading technique for the noninvasive permanent reduction of fatty bulges in the United States,^3^ results are variable^4,5^ and dependent upon the treated individual’s biological response. Partially as a result of this fact, there are several aspects of the treatment that indeed lack consensus. These include the definition of terms such as responders and non-responders, recommended time between repeat treatments of the same area, and recommendations regarding average treatments per area needed to achieve clinically meaningful results. It seems that while this study added clarity regarding several issues, some remain as unclear as ever.

When dealing with treatments that generally produce real but modest results, it can be at times challenging to define who is a non-responder. The panelists dealt with this by defining a new term, “poor responder” to refer to a patient who shows no clinically visible response but may respond to additional treatment cycles, “or alternative treatments.” I question whether this nomenclature will provide any additional clarity or benefit clinicians or patients. Based on the panel’s own definitions, it is impossible to determine that someone is a non-responder until they receive more treatments and continue to show no response. From a physiologic standpoint, it remains entirely possible that they are still having a response, but that it is so minuscule that it does not create visible results. Furthermore, if this term is adopted, I find it hard to imagine which patients would be willing to spend more money on additional treatments, having already been labeled as poor responders. Perhaps the term “slow responder” would be more appropriate.

The authors do provide clarity with regard to treatment nomenclature, differentiating between treatment cycles and treatment sessions. The former refers to each time an applicator is placed for a treatment, and the latter refers to all of the treatment cycles which occur during one patient visit.

With regard to patient evaluation and selection, there was complete consensus that the most important predictor of a successful outcome is that the applicator fits well over the treatment area, with the skin and fat to be treated filling the applicator mold. While seemingly obvious, this point cannot be overemphasized when training providers of CoolSculpting treatments. The consensus statements also wisely point out that while a visible result is the most important determinant of patient satisfaction, a comfortable patient experience during treatment is also an important factor in predicting patient satisfaction. This point is worth keeping in mind for all treatments we provide. Patients today demand not just results that match their expectations but also a positive treatment experience.

Recommendations on typical number of treatment cycles by area to achieve a visible outcome are accompanied by a reminder that these are “the minimums required for an initial visible clinical outcome… and that more cycles may be required to achieve optimal results.” The panel’s recommendations with regard to the management of AEs are reflective of the fact that plastic surgeons comprised a minority of panel members. They add nothing to what would be obvious to a junior plastic surgery resident, including that when treating contour irregularities, the determination must be made as to whether there is a volume deficit or excess, that necrosis (never once seen in my practice, which has provided over 25,000 CoolSculpting cycles to several thousand patients) requires an aggressive treatment approach, and that hyperpigmentation must be differentiated from bruising. 

I would have enjoyed seeing a recommendation that all CoolSculpting patients have photography of their markings, in addition to their standard photography. Patient weight should also be measured and recorded at each CoolSculpting visit. Similarly, I would recommend that all patients confirm in writing that applicator placement areas were reviewed with them before treatment.

In summary, this was an admirable though probably intrinsically flawed attempt to achieve consensus on many issues relating to CoolSculpting. Some of its deficiencies help emphasize the value of having plastic surgeons take a larger role in this space. It is important to note that our patients are frequently on a body contouring journey that over time may become progressively invasive. We as plastic surgeons can serve as their ideal guides on this journey, with great benefit and satisfaction to be garnered by patient and doctor alike.
